# Clinical care models in the era of next‐generation sequencing

**DOI:** 10.1002/mgg3.225

**Published:** 2016-05-12

**Authors:** Anne Slavotinek

**Affiliations:** ^1^ UCSF Benioff Children's HospitalUniversity of California, San FranciscoRoom 384D, Rock Hall, 1550 4th StSan FranciscoCalifornia94143‐2711

Personalized (or precision) medicine utilizes genomic sequence for diagnosis, identifying, and managing presymptomatic conditions, and for evaluating risks and guiding treatments (Bowdin et al. 2016). Whole exome sequencing (WES) is a critical tool for the implementation of personalized medicine and had a transformative impact on patient care in the few years since its inception as a clinical test. WES has a success rate of approximately 25% for identifying causative variants for phenotypically and genotypically heterogeneous conditions, such as intellectual disability, and is thus widely acknowledged as an effective diagnostic tool (Dixon‐Salazar et al. 2012; Biesecker and Green 2014; Lee et al. 2014; Yang et al. 2014). However, WES is distinguished from other genetic tests by the sheer number of genes that are simultaneously targeted (estimated 20,000; Biesecker and Green 2014). Controversies, such as testing options for secondary variants and their disclosure, ongoing interpretation of variants of unknown significance (VUS), and returning results in children for adult onset disorders, are abundant. In addition, the consent process for WES is complex and can cover numerous topics, encompassing basic genetics (genes, mutations), inheritance patterns, penetrance and expressivity, types of DNA variants (pathogenic, benign, VUS), incidental findings, false‐positive and false‐negative results, the scientific discoveries that may result from testing results, information privacy, nonpaternity, and the Genetic Information Nondiscrimination Act of 2008 (Bick and Dimmock 2011; Tabor et al. 2012). The informed consent process for WES in the pediatric population was first estimated to require 3–6 h (Mayer et al. 2011; Tabor et al. 2012); others have suggested that multiple encounters are needed due to the predictive component of the test and the number of potentially affected individuals (Bowdin et al. 2016). Other questions concern the incorporation of WES findings into the electronic medical record and the best method for educating health care professionals concerning the findings (Lazaridis et al. 2016).

Given these complexities and uncertainties in test provision, it is perhaps not surprising that the optimal clinical model for the incorporation of WES into patient care is not yet known. Few models for clinical delivery of WES and WGS have been published (Lazaridis et al. 2014, 2016; Bowdin et al. 2016). The first model for care delivery was established as the individualized medicine (IM) clinic at the Mayo Clinic in 2012, as part of the Center for Individualized Medicine (CIM). In this clinic, patients are referred for exome sequencing from providers from all disciplines, but permission for entry into the WES testing program is the decision of a genomic odyssey board. The IM clinic serves two service lines, comprising cancer and undiagnosed patients who have previously undergone normal genetic testing (Lazaridis et al. 2014). Patients are assigned a lead clinician, known as an IM consultant, who performs consent. All patients then attend a separate session with a genetic counselor from the CIM that covers genomic and financial counseling. After consent, sample collection, and sequencing, the variant report is interpreted at a weekly meeting of the genomic odyssey board. Recommendations are made from the board to the referring physician and the IM consultant and counselor subsequently provide the results and discuss a management plan with the patient and family (Lazaridis et al. 2014). In the first 18 months of IM clinic operations, there were 82 consultation requests and 51 patients who underwent clinical WES. A diagnosis was achieved in 15/51 cases for a positive diagnostic yield of 29% (Lazaridis et al. 2016).

The second clinic model, Sick Kids Genome Clinic, at the Hospital for Sick Children, Toronto, offers both a clinical and a research component to testing. The Sick Kids Genome Clinic aims to determine the impact of primary and secondary whole genome findings on medical management, to measure the psychosocial impact of receiving WGS results, compare total cost of WGS to arrays and panels, and to develop a data resource for future studies related to the costs and clinical consequences of genomic technologies (Bowdin et al. 2016). Participation of both parents is required, with consent or assent obtained for the child, but WGS is performed only on the child. The consent process is performed over at least two encounters that are separated by a 24‐h waiting period. Participants must agree to the active pursuit and disclosure of secondary variants, although patients and parents can select specific phenotypic categories for return of secondary findings, for example, cancer. Patients and parents can also list diseases that they do or do not wish to include for this purpose. Parents are actively contacted for results reanalysis (Bowdin et al. 2016).

At the University of California, San Francisco, we have also established a personalized medicine clinic. Since 2013, our genetics providers (two clinical geneticists and a counselor) have seen more than 100 patients that were referred from 12 providers, sometimes at the consent stage for WES, but most frequently following WES testing for results interpretation or for reanalysis (Fig. 1). Our anecdotal experience is that a separate clinic can foster a consistent approach to the process of consent and results provision, provide accurate variant interpretation and facilitate reanalysis and longitudinal data collection. However, the model of a separate clinic for WES has not systematically been compared to the efficacy of performing WES in general clinic situations, or with other nongeneticist providers. Questions that should be considered in the future include:

**Figure 1:**
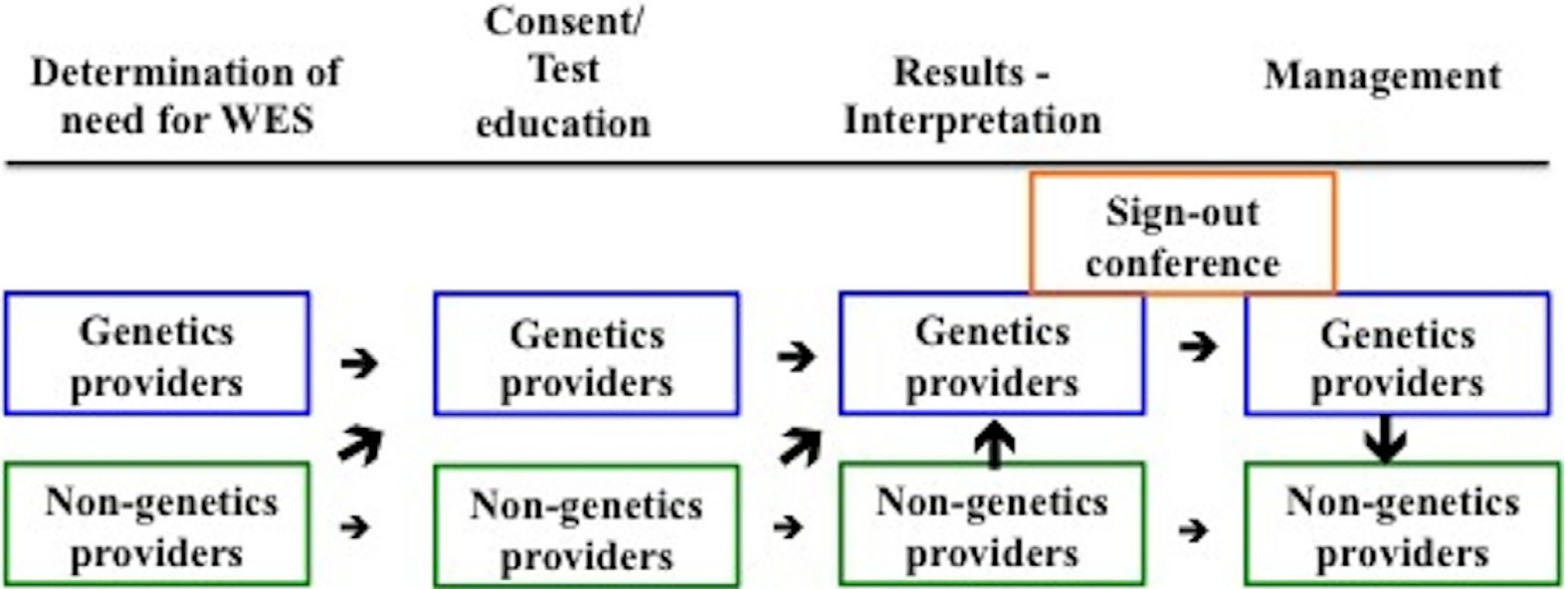
Diagram of Clinic Flow for Whole Exome Sequencing. A determination of need for whole exome sequencing (WES), consent, results provision and interpretation can be performed by Genetics or non‐Genetics health providers. However, non‐Genetics providers will frequently refer to a genetics health provider at these stages. After results are provided, management and follow‐up can also performed by either service provider, but patients are often referred back to their original clinicians. Sign‐out conferences, attended by providers from multiple disciplines, can help with accurate variant interpretation and management plans.

## Is involvement of Genetics Providers Required for WES?

Is care in a clinic staffed by genetics professionals required for optimal, or even successful, implementation of WES? For patients in whom a diagnostic result is clear, it may not be critical to involve a geneticist or counselor. However, for many individuals and families, one or more VUSs in known genes will be returned after sequencing, or the test results may list a variant in a novel gene. Each VUS and novel gene needs evaluation by the ordering clinician in order to establish clinical validity and determine the relevancy of the gene and variant to the patient's phenotype. A major source of analytic error in next‐generation sequencing is variant misinterpretation that is currently assessed using evidence gathered from published reports, in silico predictions from public databases and functional analyses, all of which can require genetics expertise (Bowdin et al. 2016). The correct interpretation of VUSs is therefore critical for deciphering WES results. Clinical geneticists, genetic counselors, and laboratory specialists are currently among the only health professionals trained in variant interpretation and thus involvement of these specialists in situations with unclear results seems reasonable. Clinicians should also be aware of the possibility of erroneous attribution of pathogenicity to a sequence variant (MacArthur et al. 2014). However, as the use of WES continues, education of all providers in variant interpretation will improve, meaning that such skills will be more widely available.

## In Which Situation is Longitudinal Follow Up Required After WES?

Some families will receive a negative exome report, without a clear result or a VUS, signifying that reanalysis of the exome data may be indicated. The extraordinary rate of new gene discovery means that reanalysis of previously negative exome data after a period as short as a year can establish a diagnosis. For example, a female with mild intellectual disability (ID) without distinguishing characteristics or dysmorphic features had WES preformed as a trio approach with biological parents that was reported as negative in 2013 (data not shown). However, in 2015, a de novo variant in a novel gene, *DDX3X*, was deemed pathogenic by the testing laboratory because of a recent publication describing mutations in this gene in association with nonsyndromic ID in females (Snijders Blok et al. 2015). Regular reanalysis of negative exome results has therefore been recommended (Biesecker and Green 2014) and longitudinal follow up should be considered for patients with negative exomes, in addition to those with VUSs for which variant interpretation may become clearer with time.

Another important argument for longitudinal care after WES is that the clinical findings associated with mutations in many genes are still evolving. There have been numerous instances of WES identifying pathogenic mutations in a gene that was previously associated with a narrower phenotype than that demonstrated by the tested individual (Beaulieu et al. 2014). Such examples of “broadening” of the phenotypic spectrum may have partially resulted from previous ascertainment bias, but are very important to recognize so that accurate counseling can be provided. For example, biallelic, causative mutations in the (breast cancer‐1)‐associated ataxia telangiectasia mutated [ATM] activator 1, or *BRAT1* gene, have been published in <20 individuals (Straussberg et al. 2015; van de Pol et al. 2015; Mundy et al. 2016). Deleterious sequence variants in this gene were thought to be associated with a severe, pharmacoresistant epileptic encephalopathy and a profound lack of developmental progress that terminated in apnea, cardiac arrest, and early death. However, recent reports have highlighted a greater clinical variability that suggests that a milder neurocognitive phenotype and longer survival are possible (Mundy et al. 2016). Delineating new syndromes and accurately recording phenotypic information over time requires longitudinal care. Such documentation may be most consistent if obtained by a single provider or clinic.

## How is the Education of Trainees and Providers Best Achieved?

As the use of WES continues to expand, trainees and providers require education about the testing indications, consent process, and how to interpret and deliver results. A clinic that offers WES on a regular basis can provide an appropriate learning environment and the opportunity for participation in the testing process for health professionals who are not familiar with next‐generation sequencing. Dedicated clinics, such as those described above, can offer these experiences together with an identity for genomic services and an opportunity to develop appropriate educational materials for the patient population served. Dedicated clinics can also systemically collect the phenotypic information and variant data that is vital to advance the field.

## Summary

The use of WES, already widespread, is likely to increase and to provide new diagnoses to many patients in whom such information has previously been unattainable. As yet, there have been few reported models of clinical care for WES despite an increasing need to consider how best to deliver this test. At present, genetics health professionals are well placed to contribute to variant interpretation and longitudinal follow up for patients undergoing next‐generation sequencing; in the future, the popularity and utility of the test may mean that a broader selection of providers will be educated to assume these responsibilities. Dedicated clinics for WES are not yet common, but appear to have several advantages that may lead to them being more widely utilized.

## Conflict of Interest

None declared.
